# The effect of glucocorticoids on serum cystatin C in identifying acute kidney injury: a propensity-matched cohort study

**DOI:** 10.1186/s12882-020-02165-1

**Published:** 2020-11-27

**Authors:** Silin Liang, Mai Shi, Yunpeng Bai, Yujun Deng, Miaoxian Fang, Jiaxin Li, Yijin Wu, Wenying Peng, Yating Hou, Heng Fang, Huidan Zhang, Chunbo Chen

**Affiliations:** 1 Department of Intensive Care Unit of Cardiovascular Surgery, Guangdong Cardiovascular Institute, Guangdong Provincial People’s Hospital, Guangdong Academy of Medical Sciences, 96 Dongchuan Road, Guangzhou, 510080 Guangdong Province People’s Republic of China; 2 Department of Critical Care Medicine, Guangdong Provincial People’s Hospital, Guangdong Academy of Medical Sciences, 106 Zhongshan Er Road, Guangzhou, 510080 Guangdong Province People’s Republic of China; 3 The Second School of Clinical Medicine, Southern Medical University, Guangzhou, China; 4 Department of Critical Medicine, Peking University Third Hospital, No.49, Huayuan Rd., Haidian District, Beijing, 100191 People’s Republic of China; 5 Center of Scientific Research, Maoming People’s Hospital, 101 Weimin Road, Maoming, 525000 Guangdong Province People’s Republic of China; 6 Department of Critical Care Medicine, Maoming People’s Hospital, 101 Weimin Road, Maoming, 525000 Guangdong Province People’s Republic of China; 7 Department of Oncology, Maoming People’s Hospital, 101 Weimin Road, Maoming, 525000 Guangdong Province People’s Republic of China

**Keywords:** Acute kidney injury, Cystatin C, Intensive care unit, Renal biomarker

## Abstract

**Background:**

Glucocorticoids may impact the accuracy of serum cystatin C (sCysC) in reflecting renal function. We aimed to assess the effect of glucocorticoids on the performance of sCysC in detecting acute kidney injury (AKI) in critically ill patients.

**Methods:**

A prospective observational cohort study was performed in a general intensive care unit (ICU). Using propensity score matching, we successfully matched 240 glucocorticoid users with 960 non-users among 2716 patients. Serum creatinine (SCr) and sCysC were measured for all patients at ICU admission. Patients were divided into four groups based on cumulative doses of glucocorticoids within 5 days before ICU admission (Group I: non-users; Group II: 0 mg < prednisone ≤50 mg; Group III: 50 mg < prednisone ≤150 mg; Group IV: prednisone > 150 mg). We compared the performance of sCysC for diagnosing and predicting AKI in different groups using the area under the receiver operator characteristic curve (AUC).

**Results:**

A total of 240 patients received glucocorticoid medication within 5 days before ICU admission. Before and after matching, the differences of sCysC levels between glucocorticoid users and non-users were both significant (*P* <  0.001). The multiple linear regression analysis revealed that glucocorticoids were independently associated with sCysC (*P* <  0.001). After matching, the group I had significantly lower sCysC levels than the group III and group IV (*P* <  0.05), but there were no significant differences in sCysC levels within different glucocorticoids recipient groups (*P* > 0.05). Simultaneously, we did not find significant differences in the AUC between any two groups in the matched cohort (*P* > 0.05).

**Conclusions:**

Glucocorticoids did not impact the performance of sCysC in identifying AKI in critically ill patients.

**Supplementary Information:**

The online version contains supplementary material available at 10.1186/s12882-020-02165-1.

## Key messages

● Glucocorticoids could induce an increase in sCysC levels. However, the relationship between the sCysC and glucocorticoids was not manifested in a dose-dependent manner.

● Glucocorticoids did not have a statistically significant effect on the performance of sCysC in detecting AKI in critically ill patients.

## Background

Acute kidney injury (AKI) is an alarming burden in critically ill patients with a high incidence and independently impacts prognosis [1–4]. The therapeutic outcome of AKI is unsatisfactory as the initiation of effective treatments are started relatively late after a noticeable elevation in serum creatinine (SCr) [5, 6]. In this context, the exploration of an early biomarker could significantly improve the prognosis of AKI.

Cystatin C (CysC), a cysteine proteinase inhibitor, is freely filtered by the glomerulus and entirely reabsorbed by the proximal tubules [7–9]. Contrast to SCr, the concentrations of serum cystatin C (sCysC) show a small individual variability as nonrenal factors only slightly influenced it [8, 9].. Therefore, sCysC may be superior to SCr in detecting a minimal change in the glomerular filtration rate (GFR) [7, 10, 11]. Previous studies showed that it might be a potential biomarker for the early detection of AKI [12–16].

However, as sCysC is widely used in clinical application, many studies reported other factors that might interfere with its concentrations beyond renal function, such as glucocorticoid therapy, thyroid function, and glycemic status [17–22]. CysC is produced by all nucleated cells in the human body at a relatively constant rate [7, 8]. However, it was shown that glucocorticoids could stimulate the production of CysC in vitro [23]. Moreover, glucocorticoid medication would also increase sCysC concentrations in experimental animals, such as rats and dogs [24, 25]. Glucocorticoids have extensive applications in human medicine because of its functions of anti-inflammation and immunosuppression. Many studies already suggested that glucocorticoids could impact sCysC concentrations independently of renal function in patients [26–29]. However, it remains undefined whether glucocorticoids influence sCysC to detect AKI in critically ill patients.

Therefore, this prospective, observational, propensity-matched study was performed in a large critically ill cohort to illuminate whether glucocorticoids impacted the diagnostic and predictive accuracy of sCysC in detecting AKI.

## Methods

### Study design and participants

This prospective observational study was undertaken in the general intensive care unit (ICU) of Guangdong Provincial People’s Hospital from October 2014 to December 2017. Patients aged 18 years or older admitted to ICU were enrolled for the study. The exclusion criteria incorporated preexisting Cushing syndrome or adrenocortical hypofunction, end-stage renal disease (ESRD) or undergoing renal replacement therapy (RRT) before admission, renal transplantation or nephrectomy, missing clinical data or refusal of consent. The primary outcome of this study was the diagnosis of AKI within 1 week after ICU admission, and the secondary outcomes included length of ICU and hospital stay, as well as ICU and hospital mortality. The protocol was followed according to that of the Strengthening the Reporting of Observational Studies in Epidemiology [30] and Standards for Reporting Diagnostic Accuracy [31] criteria. The current study received the approval of ethics committee of the Guangdong Provincial People’s Hospital and all methods were performed in accordance with the relevant guidelines and regulations. Written informed consent was obtained from each participant or a family member at the time of enrollment.

### Data collection

We prospectively collected patients’ baseline clinical data. All serum samples for determining SCr, sCysC, and serum albumin were collected concurrently within 1 h after ICU admission. SCr was measured at least once a day as a part of routine clinical care during ICU hospitalization. The following demographic and clinical characteristics were collected: age, sex, body mass index (BMI), preexisting clinical conditions, previous glucocorticoid administration within 5 days before entering ICU, admission type, baseline SCr, baseline-estimated glomerular filtration rate (eGFR), SCr at ICU admission, serum albumin and Acute Physiology and Chronic Health Evaluation II (APACHE II) score at ICU admission. The outcome variables were documented, including the occurrence of AKI within 1 week after ICU admission, length of ICU stay, length of hospital stay, ICU mortality, and in-hospital mortality. We calculated the eGFR based on the Chronic Kidney Disease Epidemiology Collaboration (CKD-EPI) creatinine equation [32].

### Definitions

AKI was diagnosed according to the Kidney Disease Improving Global Outcomes (KDIGO) criteria as any of the following: increase in SCr by 0.3 mg/dl (26.5 mmol/l) within 48 h, increase in SCr to 1.5 times of the baseline level within 1 week, or urine output < 0.5 mL/kg/h for 6 h [33]. Because of insufficient sensitivity of urine output in diuretics administration, we diagnosed AKI based on SCr [34]. We determined the baseline SCr according to the following rules ranked in descending order of preference [35]: (1) the most recent pre-ICU value between 30 and 365 days before ICU admission; (2) a stable pre-ICU value > 365 days for patients aged < 40 years (stable defined as within 15% of the lowest ICU measurement) before ICU admission; (3) pre-ICU value > 365 days before ICU admission and less than the initial SCr at ICU admission; (4) a pre-ICU value (between 3 and 39 days before ICU admission) less than or equal to the initial on-admission SCr to ICU and not distinctly during AKI; (5) the lowest SCr upon initial admission to ICU, the last ICU value, or the minimum value at follow-up to 365 days. Established AKI and later-onset AKI were diagnosed if patients reached the KDIGO criteria at ICU admission or within 1 week after ICU admission, respectively. Mild AKI was defined as KDIGO stage 1 and severe AKI was defined as stage 2 or stage 3 of KDIGO criteria [33, 36].

### Patient groups

Thus far, previous studies showed that there was a significant correlation between sCysC levels and glucocorticoids in the first 5 days after the administration of glucocorticoids [28, 29]. Therefore, the patients were divided into four groups based on accumulated doses of glucocorticoids within 5 days before admission to ICU: Group I (non-users), Group II (0 mg < prednisone ≤50 mg), Group III (50 mg < prednisone ≤150 mg), Group IV (prednisone > 150 mg). As attested by potency and duration of action of glucocorticoids, we used this dosage calculation, 4 mg/day of methylprednisolone and 0.75 mg/day of dexamethasone was equal to a dosage of 5 mg/day of prednisone [37].

### Laboratory methods

All samples were measured within 24 h after collection at the central laboratory of the Guangdong Provincial People’s Hospital. SCr, sCysC, and serum albumin were measured using a UniCel DxC 800 Synchron System (Beckman Coulter, CA, USA). sCysC was measured by immunoturbidimetry, the coefficients of inter-assay and intraassay variations for which were ≤ 5% and ≤ 10%, respectively.

### Statistical analysis

To avoid potential confounders and selection biases caused by nonrandomized in this study, we used the propensity score matching. We generated the propensity score for each patient according to the probability of receiving glucocorticoids produced by multivariable logistic regression analysis model. The multivariable logistic regression analysis model (*P* <  0.001; Hosmer and Lemeshow goodness-of-fit test, *P* = 0.935) obtained the following covariates: age, sex, BMI, APACHE II score, serum albumin, baseline eGFR, diabetes mellitus (DM), hypertension, chronic kidney disease (CKD), coronary heart disease (CHD), chronic heart failure (CHF), stroke, malignancy, chronic obstructive pulmonary disease (COPD), chronic liver disease, admission type, and grade of AKI. And then, we used the propensity score to match glucocorticoid users with non-users at a ratio of 1:4 using nearest neighbor matching. Patients without a corresponding match were excluded. After all propensity score matches were completed, we evaluated the balance in baseline variates. Propensity score matching was performed using SPSS version 23.0 (SPSS, USA, IL).

A two-tailed *P* value< 0.05 was considered statistically significant. All continuous variables did not follow normal distribution as tested by SPSS software. Continuous variables were reported as median (interquartile range, IQR) and compared using the Wilcoxon rank-sum test. Categorical variables were reported as absolute value (percentage) and compared using the chi-square test or Fisher’s exact test. The bivariate correlation analysis was used to assess the association between two variables. Multivariable linear regression analysis with a stepwise variable selection was also used to assess the relationship between sCysC and other variables. A receiver operating characteristic (ROC) curve analysis was generated, and the area under the curve (AUC) in each group as assessed using the Hanley–McNeil method, and the optimal cutoff value for AKI detection was determined with the Youden’s index. All statistical analyses were performed using SPSS version 23.0 (SPSS, IL, USA) and MedCalc version 18.2.1 (MedCalc Software, Ostend, Belgium) software programs.

## Results

### Patient characteristics and outcomes

**Fig. 1:**
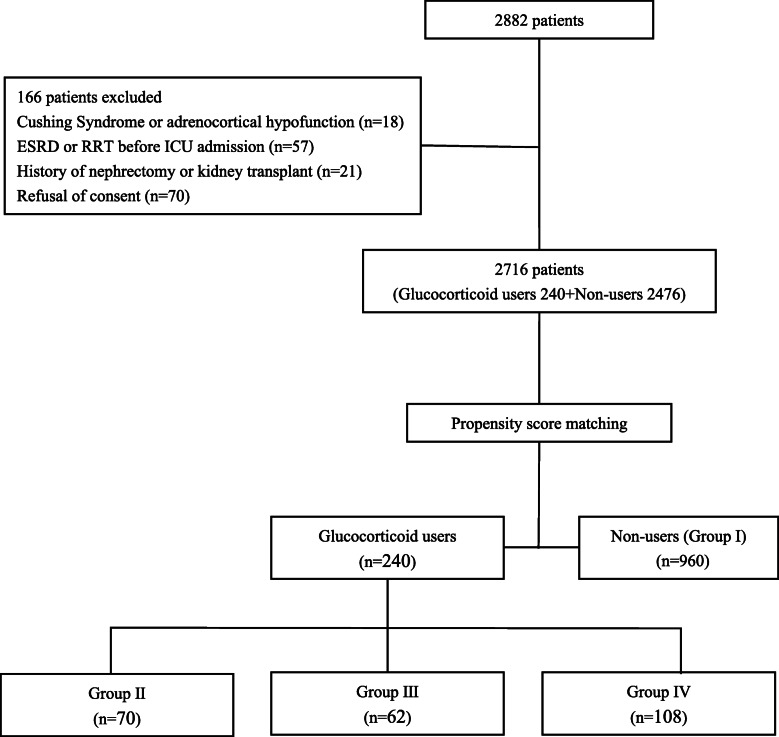
Recruitment of patients into the study. ESRD: End-stage renal disease; ICU: Intensive care unit; n: Sample size; RRT: Renal replacement therapy. Group I was defined as the cumulative doses of prednisone within 5 days before admission to ICU was equal to 0 mg. Group II was defined as the cumulative doses of prednisone within 5 days before admission to ICU were greater than 0 mg and less than or equal to 50 mg. Group III was defined as the cumulative doses of prednisone within 5 days before admission to ICU were greater than 50 mg and less than or equal to 150 mg. Group IV was defined as the cumulative doses of prednisone within 5 days before admission to ICUs were greater than 150 mg

There were 2716 patients got engaged in this study (Fig. 1). A total of 166 patients were excluded. Glucocorticoids were prescribed for 8.8% (240/2716) of patients. In total, 23.3% of patients (634/2716) met the diagnostic criteria of AKI, including patients accounted for 34.9% (221/634) occurred established AKI, and patients accounted for 65.1% (413/634) developed later-onset AKI.

Table 1 shows the characteristics of the patients. Compared with patients who were not prescribed glucocorticoids, users were more likely to have a higher APACHE II score (11 vs. 9, *P* <  0.001), and had a significantly higher percentage of comorbidities, including malignancy (27.9% vs. 14.7%, *P* <  0.001), COPD (5.4% vs. 1.8%, *P* <  0.001) and chronic liver disease (2.9% vs 1.0%, *P* = 0.017). Moreover, there was a significant difference between glucocorticoid users and non-users in admission type. Patients using glucocorticoids were more likely to suffer from more severe AKI.

**Table 1:** Baseline data and outcomes

Variables	Before matching			After matching		
	Corticosteroid users (*n* = 240)	Non-users (*n* = 2476)	*P* value	Corticosteroid users (n = 240)	Non-users (*n* = 960)	*P* value
Cumulative dose of prednisone, mg	133.3 (50.0–300.0)	0		133.3 (50.0–300.0)	0	
Baseline characteristics						
Age, years	55.0 (44.0–65.0)	55.0 (43.0–65.0)	0.949	55.0 (44.0–65.0)	54.0 (40.0–67.0)	0.800
Male sex, n (%)	158 (65.8)	1280 (51.7)	< 0.001	158 (65.8)	615 (64.1)	0.608
BMI, kg/m^2^	22.46 (20.77–24.95)	22.32 (20.70–24.44)	0.506	22.46 (20.77–24.95)	22.45 (20.95–24.46)	0.986
Preexisting clinical conditions						
Hypertension, n (%)	46 (19.2)	476 (19.2)	0.983	46 (19.2)	177 (18.4)	0.795
DM, n (%)	21 (8.8)	208 (8.4)	0.852	21 (8.8)	82 (8.5)	0.918
CKD, n (%)	12 (5.0)	90 (3.6)	0.288	12 (5.0)	45 (4.7)	0.839
CHD, n (%)	8 (3.3)	94 (3.8)	0.719	8 (3.3)	29 (3.0)	0.802
Stroke, n (%)	24 (10.0)	326 (13.2)	0.162	24 (10.0)	106 (11.0)	0.642
CHF, n (%)	8 (3.3)	62 (2.5)	0.439	8 (3.3)	27 (2.8)	0.668
Malignancy, n (%)	67 (27.9)	364 (14.7)	< 0.001	67 (27.9)	256 (26.7)	0.696
COPD, n (%)	13 (5.4)	45 (1.8)	< 0.001	13 (5.4)	35 (3.6)	0.211
Chronic liver disease, n (%)	7 (2.9)	24 (1.0)	0.017	7 (2.9)	19 (2.0)	0.372
Admission type			< 0.001			0.258
Elective surgical, n (%)	155 (64.6)	1887 (76.2)		155 (64.6)	650 (67.7)	
Emergency surgical, n (%)	17 (7.1)	205 (8.3)		17 (7.1)	84 (8.8)	
Medical, n (%)	68 (28.3)	384 (15.5)		68 (28.3)	226 (23.5)	
Baseline eGFR, ml/min/1.73 m^2^	103.70 (89.81–115.39)	100.23 (85.83–112.60)	0.036	103.70 (89.81–115.39)	103.19 (88.62–116.34)	0.875
Baseline SCr, umol/L	63.85 (54.00–76.53)	65.00 (53.36–79.69)	0.639	63.85 (54.00–76.53)	64.00 (51.60–79.50)	0.742
SCr at ICU admission umol/L	76.17 (64.00–92.15)	73.00 (59.40–91.80)	0.056	76.17 (64.00–92.15)	76.00 (60.03–96.00)	0.708
Serum albumin, g/L	30.45 (26.60–33.80)	31.60 (27.50–35.00)	0.001	30.45 (26.60–33.80)	30.30 (26.36–34.00)	0.999
APACHE II score	11 (7.0–18.0)	9 (6.0–14.0)	< 0.001	11.0 (7.0–18.0)	11 (7.0–16.0)	0.537
Primary outcomes						
Total AKI, n (%)	88 (36.7)	546 (22.1)	< 0.001	88 (36.7)	334 (34.8)	0.586
Established AKI, n (%)	29 (12.1)	192 (7.8)	0.019	29 (12.1)	128 (13.3)	0.608
Later-onset AKI, n (%)	59 (24.6)	354 (14.3)	< 0.001	59 (24.6)	206 (21.5)	0.297
Grade of AKI,			< 0.001			0.486
Non-AKI, n (%)	152 (63.3)	1930 (77.9)		152 (63.3)	626 (65.2)	
Mild AKI, n (%)	50 (20.8)	351 (14.2)		50 (20.8)	205 (21.4)	
Severe AKI, n (%)	38 (15.8)	195 (7.9)		38 (15.8)	129 (13.4)	

The non-normally distributed continuous variables are expressed as median (25th percentile to 75th percentile [interquartile range]). Categorical variables are expressed as n (%)

*APACHE II score* Acute Physiology and Chronic Health Evaluation II score; *AKI* Acute kidney injury; *BMI* Body mass index; *CKD* Chronic kidney disease; *CHD* Coronary heart disease; *CHF* Chronic heart failure; *COPD* Chronic obstructive pulmonary disease; *DM* Diabetes mellitus; *eGFR* Estimated glomerular filtration rate; *ICU* Intensive care unit; *KDIGO* Kidney Disease: Improving Global Outcomes; *n* Sample size; *SCr* Serum creatinine; Established AKI, defined as diagnosis of AKI at ICU admission; Later-onset AKI, indicated no AKI diagnosis at ICU admission but reaching the KDIGO criteria within 1 week after admission; Mild-AKI: defined as reaching KDIGO stage 1 diagnostic criteria of AKI; Severe-AKI, defined as reaching KDIGO stage 2 or stage 3 diagnostic criteria of AKI

*P* value for global comparisons among groups by rank sum test and chi-square test for continuous and categorical variables, respectively

### Factors associated with sCysC

The results of bivariate correlations analysis indicated a significant, but weak, correlation between sCysC and previous use of glucocorticoids (correlation coefficient = 0.074, *P* <  0.001) (Table 2). In multivariable linear regression analysis (Table 3), we found the same association between sCysC and previous use of glucocorticoids (standardized β = 0.092, *P* = 0.003). There was also an association between the cumulative dose of steroids and cystatin C in the above two types of correlation analysis. Besides, the correlation and regression analyses demonstrated that other factors also were associated with sCysC. Therefore, we used the propensity score matching to eliminate their impacts.

**Table 2:** Factors associated with sCysC using bivariate correlation analysis

Variables	Correlation coefficient	*P*
Spearman method		
Age, years	0.391	< 0.001
BMI, kg/m^2^	0.029	0.134
Baseline eGFR, ml/minute/1.73 m^2^	− 0.477	< 0.001
Baseline SCr, umol/L	0.438	< 0.001
SCr at ICU admission, umol/L	0.569	< 0.001
Cumulative dose of prednisone, mg	0.152	< 0.001
Serum albumin, g/L	−0.118	< 0.001
APACHE II score	0.397	< 0.001
Grade of AKI, n (%)	0.414	< 0.001
Point-biserial method		
Male sex, n (%)	0.164	< 0.001
Previous use of glucocorticoids, n (%)	0.074	< 0.001
Hypertension, n (%)	0.255	< 0.001
DM, n (%)	0.182	< 0.001
CKD, n (%)	0.473	< 0.001
CHD, n (%)	0.160	< 0.001
Stroke, n (%)	0.108	< 0.001
CHF, n (%)	0.207	< 0.001
Malignancy, n (%)	−0.003	0.889
COPD, n (%)	0.111	< 0.001
Chronic Liver disease, n (%)	0.043	0.026

*APACHE II score* Acute Physiology and Chronic Health Evaluation II score; *AKI* Acute kidney injury; *BMI* Body mass index; *CKD* Chronic kidney disease; *CHD* Coronary heart disease; *CHF* Chronic heart failure; *COPD* Chronic obstructive pulmonary disease; *DM* Diabetes mellitus; *eGFR* Estimated glomerular filtration rate; *ICU* Intensive care unit; *KDIGO* Kidney Disease: Improving Global Outcomes; *n* Sample size; *SCr* Serum creatinine; Mild-AKI: defined as reaching KDIGO stage 1 diagnostic criteria of AKI; Severe-AKI, defined as reaching KDIGO stage 2 or stage 3 diagnostic criteria of AKI

**Table 3:** Factors associated with sCysC using multivariate linear regression analysis

Independent variables	sCysC at ICU admission, mg/L	
	Standardized β	*P*
Baseline SCr, umol/L	0.002	< 0.001
SCr at ICU admission, umol/L	0.004	< 0.001
Grade of AKI	0.183	< 0.001
APACHE II score	0.009	< 0.001
Baseline eGFR, ml/min/1.73 m^2^	− 0.003	< 0.001
Admission type	−0.105	< 0.001
CKD, n (%)	0.227	< 0.001
Previous use of glucocorticoids, n (%)	0.092	0.003
Cumulative dose of prednisone, mg	0.0002	0.028
Serum albumin, g/L	−0.004	0.001
CHD, n (%)	0.118	0.001
Age	0.001	0.049
Constant	0.702 (Unstandardized)	< 0.001

*APACHE II score* Acute Physiology and Chronic Health Evaluation II score; *AKI* Acute kidney injury; *BMI* Body mass index; *CKD* Chronic kidney disease; *CHD* Coronary heart disease; *CHF* Chronic heart failure; *COPD* Chronic obstructive pulmonary disease; *DM* Diabetes mellitus; *eGFR* Estimated glomerular filtration rate; *ICU* Intensive care unit; *KDIGO* Kidney Disease: Improving Global Outcomes; *n* Sample size; *SCr* Serum creatinine; Mild-AKI: defined as reaching KDIGO stage 1 diagnostic criteria of AKI; Severe-AKI, defined as reaching KDIGO stage 2 or stage 3 diagnostic criteria of AKI

Independent variables included: baseline SCr, SCr at ICU admission, previous use of glucocorticoids, cumulative dose of prednisone, APACHE II score, grade of AKI, admission type, malignancy, stoke, hypertension, DM, CHD, CHF, COPD, chronic Liver disease, CKD, Albumin at admission, BMI, eGFR, sex, age. Variables not listed in the table were removed from the stepwise analysis. Adjusted R square 0.776

### Propensity score match

Based on the propensity score of each patient, 240 glucocorticoid users were successfully matched to 960 non-users (Table 1). There were no significant differences in the baseline characteristics between the matched glucocorticoid users and non-users.

### The concentrations of sCysC in different groups

**Fig. 2:**
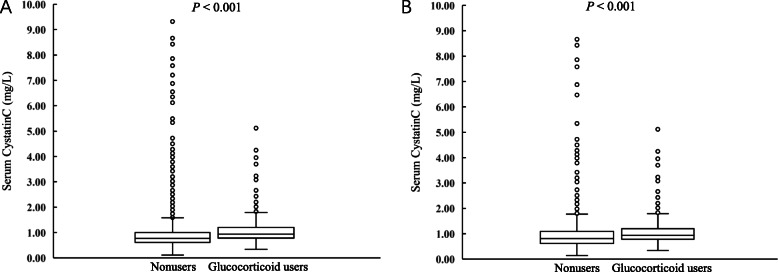
The median of sCysC levels in patients with and without glucocorticoid treatment. **a** Glucocorticoid users had higher levels of sCysC before matching (0.940 mg/L vs 0.770 mg/L; *P* <  0.001). **b** Glucocorticoid users had higher levels of sCysC after matching (0.940 mg/L vs 0.810 mg/L; *P* <  0.001)

**Fig. 3:**
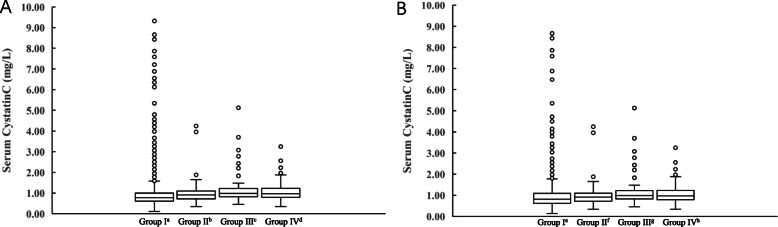
The concentration of sCysC in different groups. Grouping of standards was according to cumulative doses of glucocorticoids within 5 days before admission to ICU: Group I: non-users; Group II: 0 mg < prednisone ≤50 mg; Group III: 50 mg < prednisone ≤150 mg; Group IV: prednisone > 150 mg; **a** Before matching: a: group I vs. group II *P* <  0.05, vs. group III *P* <  0.05, and vs. group IV *P* <  0.05; b: group II vs. group I *P* < 0.05, vs. group III *P* > 0.05, and vs. group IV *P* > 0.05; c: group III vs. group I *P* < 0.05, vs. group II *P* > 0.05, and vs. group IV *P* > 0.05; d: group IV vs. group I *P* < 0.05, vs. group II *P* > 0.05, and vs. group III *P* > 0.05. **b** After matching: e: group I vs. group II *P* > 0.05, vs. group III *P* < 0.05, and vs. group IV *P* < 0.05; f: group II vs. group I *P* > 0.05, vs. group III *P* > 0.05, and vs. group IV *P* > 0.05; g: group III vs. group I *P* < 0.05, vs. group II *P* > 0.05, and vs. group IV *P* > 0.05; h: group IV vs. group I *P* < 0.05, vs. group II *P* > 0.05, and vs. group III *P* > 0.05

We found a significant difference in sCysC levels between glucocorticoid users and non-users (0.940 mg/L vs 0.810 mg/L; *P* <  0.001), although sCysC levels were elevated moderately (Fig. 2). Before matching, sCysC levels were statistically significantly lower in group I than the other three groups (*P* <  0.05), and the sCysC levels of different glucocorticoids recipient groups were not different (*P* > 0.05). After matching, there were the same results in the different glucocorticoids recipient groups (*P* > 0.05), but the sCysC levels of group I were significantly lower than the sCysC levels in group III or group IV (*P* <  0.05) (Fig. 3).

### Detection of AKI by sCysC concerning different glucocorticoids doses

Table 4 shows the AUC-ROC analysis for the performance of sCysC to detect AKI in different groups. Before matching, the AUC calculated by sCysC for the entire cohort was 0.769, 0.810, and 0.748 for total AKI, established AKI, and later-onset AKI respectively (Additional file 1: Fig. S1). After matching, the AUC calculated by sCysC for the entire cohort was 0.758, 0.802, and 0.732 for detecting total AKI, established AKI, and later-onset AKI respectively (Additional file 1: Fig. S1).

**Table 4:** Detection of AKI using sCysC by different groups

	Total AKI				Established AKI				Later-on AKI			
	AUC ROC	95%CI	Cutoff	*P*	AUC ROC	95%CI	Cutoff	*P*	AUC ROC	95%CI	Cutoff	*P*
Before matching												
Total	0.769 ± 0.011	0.753–0.785	1.00	< 0.001	0.810 ± 0.017	0.793–0.826	0.93	< 0.001	0.748 ± 0.014	0.730–0.765	1.02	< 0.001
Group I	0.769 ± 0.012	0.752–0.786	1.00	< 0.001	0.810 ± 0.018	0.793–0.827	0.93	< 0.001	0.747 ± 0.015	0.729–0.765	1.02	< 0.001
Group II	0.733 ± 0.060	0.613–0.831	1.00	< 0.001	0.760 ± 0.086	0.622–0.868	1.04	0.003	0.714 ± 0.072	0.580–0.825	1.00	0.003
Group III	0.736 ± 0.064	0.609–0.840	1.21	< 0.001	0.781 ± 0.085	0.631–0.891	1.48	0.001	0.712 ± 0.078	0.569–0.829	1.09	0.007
Group IV	0.724 ± 0.061	0.629–0.805	1.36	< 0.001	0.779 ± 0.077	0.676–0.862	0.99	< 0.001	0.707 ± 0.074	0.608–0.793	1.64	0.005
After matching												
Total	0.758 ± 0.015	0.733–0.782	1.09	< 0.001	0.802 ± 0.021	0.775–0.827	0.98	< 0.001	0.732 ± 0.019	0.704–0.759	1.09	< 0.001
Group I	0.768 ± 0.017	0.740–0.794	0.95	< 0.001	0.812 ± 0.023	0.782–0.839	0.98	< 0.001	0.740 ± 0.021	0.709–0.770	0.95	< 0.001
Group II	0.733 ± 0.060	0.613–0.831	1.00	< 0.001	0.760 ± 0.086	0.622–0.868	1.04	0.003	0.714 ± 0.072	0.580–0.825	1.00	0.003
Group III	0.736 ± 0.064	0.609–0.840	1.21	< 0.001	0.781 ± 0.085	0.631–0.891	1.48	0.001	0.712 ± 0.078	0.569–0.829	1.09	0.007
Group IV	0.724 ± 0.061	0.629–0.805	1.36	< 0.001	0.779 ± 0.077	0.676–0.862	0.99	< 0.001	0.707 ± 0.074	0.608–0.793	1.64	0.005

*95% CI* 95% Confidence interval; *AKI* Acute kidney injury; *AUC-ROC* Area under the receiver operating characteristic curve; *ICU* Intensive care unit; *KDIGO* Kidney Disease: Improving Global Outcomes; Established AKI, defined as diagnosis of AKI at ICU admission; Later-onset AKI, indicated no AKI diagnosis at ICU admission but reaching the KDIGO criteria within 1 week after admission;

Grouping of standards was according to cumulative doses of glucocorticoids within 5 days before admission to ICU: Group I: non-users; Group II: 0 mg < prednisone ≤50 mg; Group III: 50 mg < prednisone ≤150 mg; Group IV: prednisone > 150 mg;

Before matching: Total AKI: AUC of Group I vs. AUC of Group II, Z = 0.588, *P* = 0.556. AUC of Group I vs. AUC of Group III, Z = 0.507, *P* = 0.612. AUC of Group I vs. AUC of Group IV, Z = 0.724, *P* = 0.469. AUC of Group II vs. AUC of Group III, Z = −0.034, *P* = 0.973. AUC of Group II vs. AUC of Group IV, Z = 0.105, *P* = 0.916. AUC of Group III vs. AUC of Group IV, Z = 0.136, *P* = 0.892. Established AKI: AUC of Group I vs. AUC of Group II, Z = 0.569, *P* = 0.569. AUC of Group I vs. AUC of Group III, Z = 0.334, *P* = 0.739. AUC of Group I vs. AUC of Group IV, Z = 0.392, *P* = 0.695. AUC of Group II vs. AUC of Group III, Z = − 0.174, *P* = 0.862. AUC of Group II vs. AUC of Group IV, Z = − 0.165, *P* = 0.869. AUC of Group III vs. AUC of Group IV, Z = 0.017, *P* = 0.986. Later-on AKI: AUC of Group I vs. AUC of Group II, Z = 0.449, *P* = 0.654. AUC of Group I vs. AUC of Group III, Z = 0.441, *P* = 0.660. AUC of Group I vs. AUC of Group IV, Z = 0.530, *P* = 0.596. AUC of Group II vs. AUC of Group III, Z = 0.019, *P* = 0.985. AUC of Group II vs. AUC of Group IV, Z = 0.068, *P* = 0.946. AUC of Group III vs. AUC of Group IV, Z = 0.047, *P* = 0.963

After matching: Total AKI: AUC of Group I vs. AUC of Group II, Z = 0.561, *P* = 0.575. AUC of Group I vs. AUC of Group III, Z = 0.483, *P* = 0.629. AUC of Group I vs. AUC of Group IV, Z = 0.695, *P* = 0.487. AUC of Group II vs. AUC of Group III, Z = − 0.034, *P* = 0.973. AUC of Group II vs. AUC of Group IV, Z = 0.105, *P* = 0.916. AUC of Group III vs. AUC of Group IV, Z = 0.136, *P* = 0.892. Established AKI: AUC of Group I vs. AUC of Group II, Z = 0.584, *P* = 0.559. AUC of Group I vs. AUC of Group III, Z = 0.352, *P* = 0.725. AUC of Group I vs. AUC of Group IV, Z = 0.411, *P* = 0.681. AUC of Group II vs. AUC of Group III, Z = − 0.174, *P* = 0.862. AUC of Group II vs. AUC of Group IV, Z = − 0.165, *P* = 0.869. AUC of Group III vs. AUC of Group IV, Z = 0.017, *P* = 0.986. Later-on AKI: AUC of Group I vs. AUC of Group II, Z = 0.347, *P* = 0.729. AUC of Group I vs. AUC of Group III, Z = 0.347, *P* = 0.729. AUC of Group I vs. AUC of Group IV, Z = 0.429, *P* = 0.668. AUC of Group II vs. AUC of Group III, Z = 0.019, *P* = 0.985. AUC of Group II vs. AUC of Group IV, Z = 0.068, *P* = 0.946. AUC of Group III vs. AUC of Group IV, Z = 0.047, *P* = 0.963

The patients were split into four groups based on whether they received glucocorticoid therapy and the accumulated doses of glucocorticoids within 5 days before ICU admission. Before matching, the AUC for sCysC in detecting total AKI was 0.769, 0.733, 0.736 and 0.724 in group I, group II, group III, and group IV respectively. The AUC for sCysC in diagnosing established AKI was 0.810, 0.760, 0.781, and 0.779 in group I, group II, group III, and group IV respectively. The AUC for sCysC in predicting later-onset AKI was 0.747, 0.714, 0.712, 0.707 in group I, group II, group III, and group IV respectively. After matching, the AUC for sCysC in detecting total AKI, established AKI, and later-onset AKI was 0.768, 0.812, and 0.740 respectively in group I; 0.733, 0.760, and 0.714 respectively in group II; 0.736, 0.781, and 0.712 respectively in group III; 0.724, 0.779, and 0.707 respectively in group IV. In summary, we did not observe a significant difference between AUC in any two groups for total AKI, established AKI, and later-onset AKI before and after the matching. Before and after matching, the cutoff values of sCysC in detecting AKI were different in diverse glucocorticoids recipient groups, but the change of cutoff values of sCysC were irregular.

## Discussion

In this prospective, propensity-matched cohort study, we evaluated whether glucocorticoids affect the ability of sCysC to detect AKI in critically ill patients. We adopted a propensity score matching to eliminate the influences of potential confounding factors. Before and after matching, we found that the abilities of sCysC in detecting AKI were not different among patients with or without glucocorticoid therapy.

sCysC has an advantage that it might be less influenced by nonrenal factors than SCr [8, 9]. However, many studies have shown conflicting results. These studies suggested that the relationship between GFR and sCysC might have a potential variability due to some nonrenal factors, including age, sex, thyroid hormones, glycemic status, hepatic impairment, as well as glucocorticoids [17–22, 26–29, 38–43]. Bjarnadottir et al. demonstrated in vitro that dexamethasone could lead to CysC level increase by the dose-dependent manner [23]. Recent studies confirmed this in animals [24, 25], and we found similar results in our current study. We found that sCysC level in glucocorticoid users was higher than that in non-users, no matter with or without matching. This phenomenon might be caused by glucocorticoids increasing the transcription of the CysC by induction of the promoter [23]. Alternatively, glucocorticoids have a nephrotoxic effect as its ability of causing metabolic alkalosis and hypertension [44]. This may be another reason why glucocorticoids could lead to an increase in the sCysC level.

Numerous previous clinical studies verified the relationship between sCysC level and glucocorticoids in patients. Cimerman et al. found that sCysC levels were elevated in steroid-independent patients with asthma after 1 week of methylprednisolone treatment [18]. Several studies in renal transplant recipients have also shown that sCysC levels are correlated with glucocorticoids [26–29, 39]. Risch et al. showed that the level of sCysC are positively correlated with the doses of glucocorticoids [26]. However, their study suggested that glucocorticoids did not affect the detection of impaired renal function by sCysC, as their obtained data indicated that sCysC was still more accurate than SCr to assess the renal function in renal transplant patients. Similarly, Pöge et al. found that the highest sCysC levels occurred on day 2 with high-doses of glucocorticoids in renal transplant patients, which suggested changes in sCysC levels develop fast [27]. Furthermore, Mendiluce et al. noticed that the increase of sCysC levels coincides with high-doses glucocorticoid treatment in the first 5 days after renal transplantation and then decreased [28]. Such studies suggested that the change of sCysC levels might be reversible. Some studies further demonstrated that sCysC could underestimate GFR resulted from this association [26, 39, 45]. Le Bricon et al. suggested that sCysC underestimated GFR in 14% in 3 months after renal transplantation [45]. Therefore, sCysC may be of limited value in assessing renal function in the case of using glucocorticoids due to underestimating of GFR. However, a recent study by Silva et al. did not observe such interference in lupus nephritis patients undergoing glucocorticoid therapy [46]. They speculated that the gene that codifies CysC was already activated by the induction because lupus nephritis patients used glucocorticoid medication for a long time. Another study had a discrepant conclusion from a study performed by Cimerman et al. in patients with asthma. However, the glucocorticoids treatment is provided by inhalation, but not systemic use [47]. Previous studies also supported that glucocorticoids not only could raise sCysC levels but also are associated in a dose-dependent manner with increased sCysC [26, 39]. In the present study, a multiple linear regression analysis showed that glucocorticoid uses were independently correlated with sCysC. After using the statistical approach of propensity score matching, we demonstrated that the elevation of sCysC levels was related to higher doses of glucocorticoids. However, we did not observe a dose-dependent relationship between the doses of glucocorticoids and sCysC levels. Accumulating evidences suggest that glucocorticoids may have a role in interfering with sCysC concentrations. Therefore, we can not exclude the possibility that using glucocorticoids may impact the value of sCysC as a marker of AKI.

As previous and present studies suggested, glucocorticoids induce an elevation of sCysC levels. So, we designed this study to solve the problem of whether the glucocorticoids could impact the performance of sCysC in detecting AKI. The results suggested that glucocorticoids had no statistically significant effect on sCysC in diagnosing and predicting AKI. We suspect that this phenomenon might be a result of the increased sCysC caused by glucocorticoids is only temporary and finite.

This study was not without limitations. First, we used propensity score matching to minimize the effects of potential confounders and selection bias caused by the nonrandom allocation to either the glucocorticoid users or non-users group. Nevertheless, this approach might still have limitations in balancing these factors between the two groups*.* Second, because our study was not an interventional trial, and we measured sCysC only once at ICU admission, we could not evaluate how long after withholding glucocorticoid therapy sCysC levels are affected. Third, this study did not reveal the rule of change of sCysC cutoff value for AKI detection when patients received glucocorticoid medication. We thought a larger sample size of glucocorticoid users was needed to reveal this rule.

## Conclusion

Glucocorticoids did not impact the performance of sCysC in identifying AKI in critically ill patients, although the levels of sCysC might be slightly affected by glucocorticoid treatment.

## Supplementary Information

**Additional file 1:Fig. S1** Performance of sCysC for AKI detection before and after matching. **A** Performance of sCysC for total AKI detection before and after matching; **B** Performance of sCysC for established AKI detection before and after matching; **C** Performance of sCysC for later-onset AKI detection before and after matching; Established AKI, defined as diagnosis of AKI at ICU admission; Later-onset AKI, indicated no AKI diagnosis at ICU admission but reaching the KDIGO criteria within 1 week after admission; AKI: Acute kidney injury; ICU: Intensive care unit; KDIGO: Kidney Disease: Improving Global Outcomes;

## Data Availability

The datasets generated and/or analyzed during this study are not publicly available, owing to currently ongoing research studies, but the data are available from the corresponding author on reasonable request.

## Abbreviations

95% CI: 95% Confidence interval
AKI: Acute kidney injury
APACHE II: Acute physiology and chronic health evaluation II score
AUC: Area under the receiver operating characteristic curve
BMI: Body mass index
CHD: Coronary heart disease
CHF: Chronic heart failure
CKD: Chronic kidney disease
COPD: Chronic obstructive pulmonary disease
CysC: Cystatin C
DM: Diabetes mellitus
eGFR: Estimated glomerular filtration rate
EPI: Epidemiology collaboration
ESRD: End-stage renal disease
GFR: Glomerular filtration rate
ICU: Intensive care unit
KDIGO: Kidney disease: improving global outcomes
n: Sample size
ROC: Receiver operating characteristic curve
RRT: Renal replacement therapy
SCr: Serum creatinine
sCysC: Serum cystatin C
