# Supplementary figures and images for: The effect of glucocorticoids on serum cystatin C in identifying acute kidney injury: a propensity-matched cohort study
Source: BMC Nephrol. 2020 Nov 27;21:519. doi: 10.1186/s12882-020-02165-1 (PMC7694927; doi:10.1186/s12882-020-02165-1)

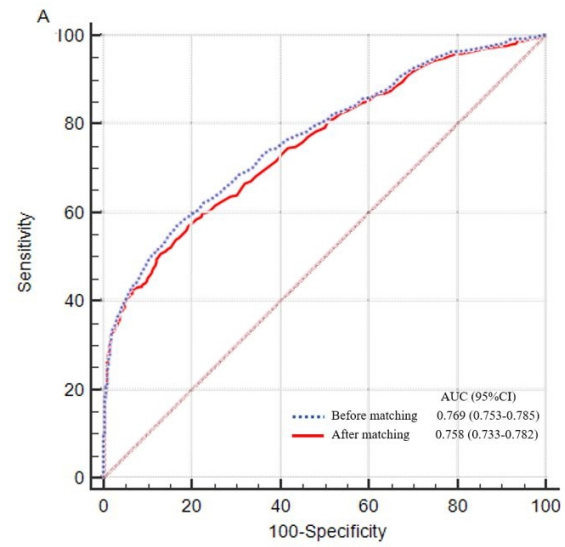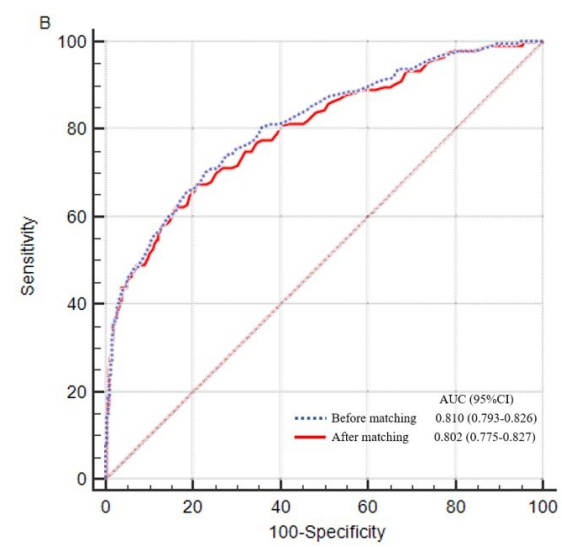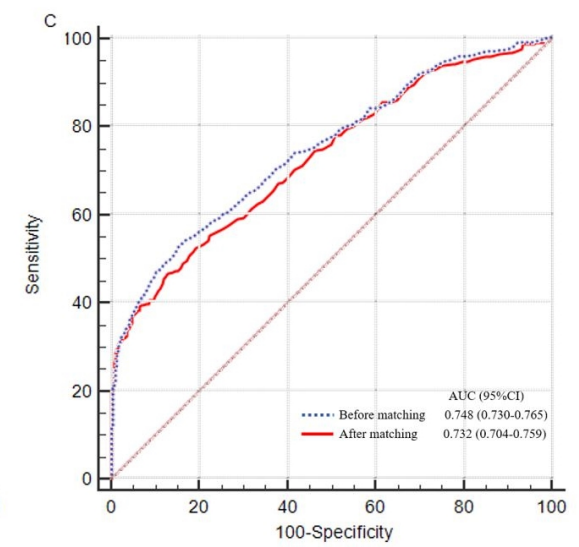

Supplement: Supplementary file 1 — Additional file 1:Fig. S1 Performance of sCysC for AKI detection before and after matching. A Performance of sCysC for total AKI detection before and after matching; B Performance of sCysC for established AKI detection before and after matching; C Performance of sCysC for later-onset AKI detection before and after matching; Established AKI, defined as diagnosis of AKI at ICU admission; Later-onset AKI, indicated no AKI diagnosis at ICU admission but reaching the KDIGO criteria within 1 week after admission; AKI: Acute kidney injury; ICU: Intensive care unit; KDIGO: Kidney Disease: Improving Global Outcomes; [file 12882_2020_2165_MOESM1_ESM.pdf]
